# Effectiveness of Electroconvulsive Therapy in Postpartum Psychosis: A Systematic Review

**DOI:** 10.1192/j.eurpsy.2025.2123

**Published:** 2025-08-26

**Authors:** O. Herrera-Barrón, X. Cors-Cepeda, C. López-García, J. Paez-Mayorga

**Affiliations:** 1 School of Medicine and Health Sciences at Tecnológico de Monterrey, Monterrey, Mexico

## Abstract

**Introduction:**

Postpartum psychosis (PPP) is a psychiatric condition that arises shortly after childbirth. Electroconvulsive therapy (ECT) offers rapid symptom relief, particularly in severe cases. Despite reports of ECT effectiveness in PPP, its use remains limited and unstandardized.

**Objectives:**

This systematic review aims to evaluate the clinical effectiveness of ECT in treating PPP.

**Methods:**

The electronic databases PubMed/MEDLINE, Cochrane, SciELO, SCOPUS, and WOS were screened for studies reporting ECT outcomes in PPP following the Preferred Reporting Items for Systematic Reviews and Meta-Analyses guidelines. Studies published between 2004 and 2024, written in English, and focused on subjects up to 12 months postpartum were included. All studies were vetted by ≥ 2 reviewers. Bias was assessed with the JBI Critical Appraisal Tool.

**Results:**

A total of 255 studies were identified and 7 met the inclusion criteria. ECT was exclusively used in severe PPP refractory to pharmacological intervention. Symptom improvement was reported in 100% of cases and most achieved total remission. Total ECT sessions ranged from 5 to 15, with symptom improvement after 1-6 sessions and remission after 5-11 sessions. ECT side effects were transient and included memory loss, mild cognitive deficit, and pain.

**Conclusions:**

High remission rates on ECT were demonstrated where pharmacological intervention was insufficient, highlighting its effectiveness as a rapid and safe intervention for PPP. Adverse effects were transient and manageable, underscoring ECT safety. Small sample sizes and variability in ECT protocols limit the generalizability of the findings. Further evidence from prospective studies is needed to consider ECT as a first line treatment for PPP.

**Disclosure of Interest:**

None Declared

